# The role of ginkgo leaf extract and dipyridamole adjuvant treatment for reduction of lower limb deep vein thrombosis after proximal femoral nail antirotation surgery in elderly patients with intertrochanteric femoral fracture

**DOI:** 10.3389/fphar.2026.1822449

**Published:** 2026-05-26

**Authors:** Jianbo Xu, Bo Li

**Affiliations:** 1 Department of Rehabilitation Medicine, Yantaishan Hospital, Yantai, China; 2 Department of Orthopedic Trauma, The Second Affiliated Hospital of Shandong First Medical University, Tai’an, China

**Keywords:** deep vein thrombosis, dipyridamole, ginkgo leaf extract, intertrochanteric femoral fracture, proximal femoral nail antirotation, thrombosis

## Abstract

**Introduction:**

Postoperative lower limb deep vein thrombosis (DVT) remains a critical complication following proximal femoral nail antirotation (PFNA) surgery for intertrochanteric femoral fracture (IFF) in the elderly. This study aimed to assess the efficacy of adjuvant Ginkgo Leaf Extract and Dipyridamole (GLED) therapy in reducing DVT incidence.

**Methods:**

This retrospective cohort study included elderly patients with intertrochanteric fractures treated with PFNA between August 2018 and July 2022. Patients were divided into a conventional group receiving rivaroxaban and a GLED group receiving rivaroxaban plus GLED. Evaluations included DVT incidence (postoperative days 0–7, 8–14, 15–21) and measurements of coagulation parameters, hemorheological parameters, and inflammatory markers at 8 h and 7 days post-surgery.

**Results:**

The analysis involved 227 patients (126 conventional, 101 GLED). The DVT incidence in the GLED group was significantly lower at 8–14 days (4.08% vs. 12.71%; p = 0.047) and 15–21 days (1.06% vs. 11.65%; p = 0.007). At 7 days, the GLED group showed significantly prolonged activated partial thromboplastin time (31.75 s vs. 31.24 s; p = 0.020) and prothrombin time (13.75 s vs. 13.52 s; p = 0.012), lower fibrinogen (3.18 g/L vs. 3.85 g/L; p < 0.001) and D-dimer (225.41 μg/L vs. 338.74 μg/L; p < 0.001), reduced blood viscosity (low shear: 13.14 vs. 15.26 mPa·s; p < 0.001), and decreased inflammatory markers (Interleukin-8: 21.29 vs. 22.62 pg/mL; p = 0.002).

**Conclusion:**

Adjuvant GLED therapy is associated with a significantly lower subacute DVT incidence after PFNA in this retrospective cohort. Further prospective studies are required to confirm a causal relationship.

## 1 Introduction

Intertrochanteric femoral fracture (IFF) is a common debilitating injury among the elderly population, typically caused by low-energy trauma such as falls (Ando et al., 2024). Clinically, IFF is characterized by persistent hip pain, restricted mobility, and severe functional impairment (Kürüm et al., 2023). Due to global aging trends and an increase in osteoporosis, the incidence of IFF has sharply risen, imposing a significant economic burden on society and healthcare systems (Wittauer et al., 2025). Surgical intervention, mainly proximal femoral nail antirotation (PFNA), offers advantages such as small incisions, minimal blood loss, and early functional rehabilitation (Nguyen et al., 2024). However, postoperative deep vein thrombosis (DVT) in the lower extremities remains a dreaded complication due to venous stasis, hypercoagulability, and endothelial injury triggered by trauma and surgery, posing risks of pulmonary embolism and chronic venous insufficiency (Xiang et al., 2022). It not only prolongs hospital stays and increases medical expenses but also poses a high risk of fatal pulmonary embolism (Li et al., 2024). Pharmacological thromboprophylaxis, primarily using direct oral anticoagulants like rivaroxaban, is commonly adopted (Wen et al., 2025). Despite its efficacy, the incidence of DVT persists, indicating limitations of monotherapy in fully combating the multifactorial pathogenesis of thrombosis, which necessitates exploring adjunctive strategies.

New evidence highlights the critical role of postoperative inflammatory response and hemorheological disturbances in the development of DVT. Inflammatory cytokines can activate the coagulation cascade, promoting a prothrombotic endothelial phenotype (Shao et al., 2025). Meanwhile, increased blood viscosity and impaired microcirculation lead to venous stasis (Bettiol et al., 2023). Ginkgo leaf extract, a traditional Chinese medicine containing flavonoids and terpenoids, possesses potent antioxidant, anti-inflammatory, and antiplatelet aggregation properties (Yu et al., 2025). Dipyridamole, an antiplatelet drug widely used for preventing cerebrovascular events, has been shown to improve microcirculation (Yan et al., 2025). The combination of ginkgo leaf extract and dipyridamole (GLED) has been studied for its potential to inhibit platelet activation, reduce blood viscosity, and enhance endothelial function (Liu et al., 2024). While several studies have demonstrated the effectiveness of GLED in reducing postoperative DVT in various surgical settings, there is limited data specifically addressing its role in elderly patients undergoing PFNA surgery for IFF.

This study aims to evaluate the efficacy of GLED as an adjuvant therapy in reducing the incidence of lower extremity DVT after PFNA surgery in elderly IFF patients. By analyzing coagulation parameters, hemorheological indicators, and inflammation markers, we aim to elucidate the underlying mechanisms of GLED’s protective effects. The novelty lies in investigating the multifaceted potential of this specific compound in a high-risk orthopedic setting. The results of this study may provide valuable clinical evidence for optimizing postoperative anticoagulation protocols and improving patient outcomes in this high-risk population.

## 2 Materials and methods

### 2.1 Research design

**FIGURE 1:**
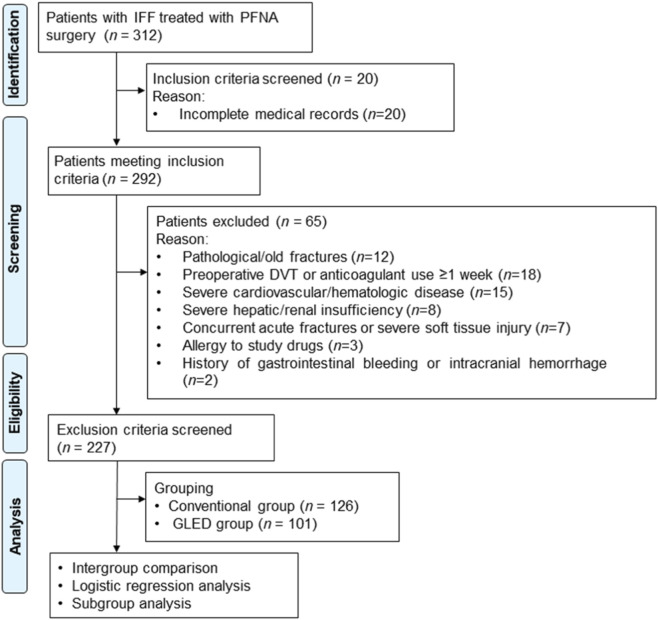
Patient selection flow diagram. IFF, Intertrochanteric Femoral Fracture; PFNA, Proximal Femoral Nail Antirotation; DVT, Deep Vein Thrombosis; GLED, Ginkgo Leaf Extract and Dipyridamole.

This study is a retrospective cohort study that selected elderly patients diagnosed with IFF and treated with PFNA surgery in our hospital’s orthopedics department from August 2018 and July 2022 (Figure 1). Inclusion criteria were: (1) age ≥60 years; (2) definitive diagnosis of IFF through imaging examinations (Green, 2010); (3) Evans fracture classification types III to V (Evans and Volume, 1949); (4) complete medical records, including preoperative baseline data, surgical records, postoperative treatment plans, laboratory test results, and follow-up data. Exclusion criteria were: (1) pathological fractures, old fractures; (2) preoperative diagnosis of lower limb deep vein thrombosis (DVT) confirmed by color Doppler ultrasound or regular use of anticoagulant drugs for ≥1 week; (3) comorbid severe cardiovascular diseases, hematological diseases, severe hepatic and renal insufficiency, nephrotic syndrome, organic lesions of the lower limb vessels, or lower limb motor dysfunction; (4) concurrent acute fractures at other sites or severe soft tissue injuries; (5) allergic to rivaroxaban, GLED injection, and related components; (6) history of gastrointestinal bleeding or intracranial hemorrhage.

Based on the differences in postoperative anticoagulation treatment protocols, 227 patients who met the inclusion and exclusion criteria were defined into a conventional group (n = 126) and a GLED group (n = 101). The conventional group was defined as receiving only standard rivaroxaban anticoagulation therapy postoperatively without additional auxiliary drugs that could affect thrombosis. The GLED group was defined as receiving additional GLED adjuvant therapy on top of the standard rivaroxaban anticoagulation therapy provided to the conventional group. The treatment duration for both groups was 30 days. The patient groups in this study were based on the actual treatment regimens received during clinical practice, reflecting the natural grouping characteristic of retrospective studies using real-world data. The difference in sample sizes between the two groups resulted from the number of eligible patients who received different treatment protocols during the study period, rather than random assignment. All treatment decisions were made by clinicians prior to the start of the study, according to the treatment guidelines and individual patient conditions at that time.

### 2.2 Ethical statement

This retrospective clinical study’s protocol was reviewed and approved by the Medical Ethics Committee of The Second Affiliated Hospital of Shandong First Medical University, which issued the ethic approval (Approval Number: 2022-079). The study was conducted strictly in accordance with the Declaration of Helsinki (revised in 2013) and relevant medical ethics standards in our country. Study data were extracted in compliance with the hospital’s electronic medical record system, and all included patients’ medical records, laboratory test results, surgical records, and follow-up data were objective medical information formed during routine clinical care. Given the retrospective design of this study, which did not interfere with the original treatment plans of patients and did not increase any additional medical risks or financial burdens to patients during the data extraction process, the requirement for obtaining informed consent was waived after approval by the ethics committee.

### 2.3 Treatment procedures

#### 2.3.1 Proximal femoral nail antirotation surgery

Both groups of patients underwent PFNA surgery performed by the same team of experienced orthopedic surgeons. Patients received continuous epidural anesthesia, and antibiotics were administered 0.5 h before surgery to prevent infection. Patients were placed in a supine position with the affected limb in a neutral position, adducted at 15°. Closed reduction was performed under fluoroscopic guidance, followed by a 5 cm incision made above the greater trochanter. The guide wire was inserted approximately 0.5 cm medial to the apex of the greater trochanter. After the guide wire was introduced into the medullary canal, reaming was performed using a hollow drill. An appropriately sized PFNA main nail was then inserted into the medullary canal, the guide wire was removed, and the depth and anteversion angle of the main nail were adjusted. A threaded guide wire was placed, and its position was confirmed using fluoroscopy. After measuring the appropriate length for the helical blade, it was hammered in and locked. Depending on the fracture pattern and bone quality, distal locking screws were selected and inserted. Once satisfactory fixation was confirmed under fluoroscopy, the end cap was installed, hemostasis was achieved, a drainage tube was placed, and the layers were closed sequentially.

#### 2.3.2 Postoperative anticoagulation treatment

1. Conventional Anticoagulation Treatment: Rivaroxaban tablets (Approval No. HJ20181081, Bayer AG, Germany) were orally administered starting 8 h post-surgery, once daily at a dose of 10 mg, for a duration of 30 days.
2. GLED Adjuvant Therapy: Starting from the first postoperative day, GLED injection (Approval No. H52020031, Guizhou Yibai Pharmaceutical Co., Ltd., Guizhou Province) was administered intravenously, once daily at a dose of 20 mL, for a duration of 30 days.

### 2.4 Evaluation indicators

#### 2.4.1 Primary evaluation indicator

The diagnosis of lower limb DVT was based on color Doppler ultrasound findings according to established diagnostic criteria. Ultrasound criteria for DVT included: (1) non-compressibility of the vein; (2) intraluminal thrombus visualized as echogenic material; (3) absence or reduction of spontaneous blood flow on Doppler imaging; and (4) absence of respiratory phasicity or loss of augmentation with distal compression (Liew et al., 2012). Bilateral lower extremity venous color Doppler ultrasound examinations for all patients were performed and reported by a fixed physician from our hospital’s ultrasound department, who had over 5 years of experience in vascular ultrasound diagnosis. The examinations followed a standardized scanning protocol and diagnostic criteria. The examination frequency was as follows: all patients underwent scheduled bilateral lower extremity venous ultrasound screenings on postoperative days 1, 3, 7, 14, and 21. Additionally, if patients exhibited any clinical symptoms or signs (leg swelling, pain, tenderness, Homan’s sign, etc.) suggestive of DVT during follow-up, additional ultrasound examinations were conducted as needed. The DVT incidence rate (%) was calculated as the number of patients diagnosed with DVT during the corresponding period divided by the total number of patients in the group, multiplied by 100%. The incidence of lower limb DVT was recorded within three time periods: postoperative, immediate to 7 days, 8–14 days, and 15–21 days. DVT incidence rates for each time period (0–7 days, 8–14 days, 15–21 days) represent the proportion of patients who were free of DVT at the beginning of that period and developed a first DVT event during the period. Patients who developed DVT in an earlier period were excluded from the denominator for subsequent periods. Thus, the reported rates are period-specific incident risks, not cumulative incidences. The postoperative period was divided into three time intervals (days 0–7, 8–14, and 15–21) based on the typical evolution of thrombotic risk after major orthopedic surgery: early (dominated by surgical trauma and immobilization), subacute (peak inflammation and mobilization), and late subacute (ongoing hypercoagulability). These intervals allow assessment of the temporal pattern of DVT occurrence and the differential efficacy of GLED across phases.

#### 2.4.2 Secondary evaluation indicators

Coagulation parameters, hemorheological parameters, and inflammatory markers were collected at 8 h post-surgery (baseline before anticoagulation therapy) and at 7 days post-surgery. Activated partial thromboplastin time (APTT), prothrombin time (PT), fibrinogen (FIB), and D-dimer (D-D) were measured using an automated coagulation analyzer (CA2000i, Sysmex, Japan). Whole blood viscosity (high shear viscosity [HS], medium shear viscosity [MS], low shear viscosity [LS]) and plasma viscosity (PV) were measured using a fully automatic blood viscometer (990BT3, Chongqing Nanfang Numerical Control Equipment Co., Ltd., China). High-sensitivity C-reactive protein (hs-CRP) was measured using a fully automatic chemiluminescence immunoassay analyzer (Cobas e801, Roche Diagnostics, Switzerland). Tumor necrosis factor-alpha (TNF-α) and interleukin-8 (IL-8) were measured using the Inflammation 20-Plex Human ProcartaPlex™ Kit (EPX20-12185-901, Thermo Fisher Scientific, United States). Additionally, the occurrence of anticoagulation-related adverse reactions within 30 days post-surgery was recorded. Major bleeding was defined according to International Society on Thrombosis and Haemostasis (ISTH) criteria as: (1) fatal bleeding; (2) symptomatic bleeding in a critical area or organ (intracranial, intraspinal, intraocular, retroperitoneal, intra-articular, pericardial, or intramuscular with compartment syndrome); (3) bleeding causing a fall in hemoglobin level of ≥20 g/L (≥2 g/dL) or leading to transfusion of ≥2 units of packed red blood cells or whole blood. Minor bleeding included gingival bleeding, skin ecchymosis, and other non-serious bleeding events not meeting major criteria. Gastrointestinal symptoms (nausea, vomiting) were also recorded as non-bleeding adverse events. In this study, the main bleeding events observed were gum bleeding and skin bruising, both of which were minor bleeding incidents. No major bleeding events, as defined by international standards, occurred.

### 2.5 Statistical analysis

This study utilized SPSS 29.0 statistical software (SPSS Inc., Chicago, IL, United States) for statistical analysis. A two-tailed significance level of α = 0.05 was set, with P < 0.05 considered statistically significant. Initially, the Shapiro-Wilk test was used to assess the normality of all continuous variables, which were confirmed to follow a normal distribution (P > 0.05 for all continuous variables) and are presented as mean ± standard deviation (M ± SD). Independent samples t-tests were employed for between-group comparisons of continuous variables. For continuous variables measured at two time points (postoperative 8 h and 7 days), two-way repeated-measures analysis of variance (ANOVA) was performed to assess the main effects of Group (conventional vs. GLED), Time (8 h vs. 7 days), and the Group × Time interaction. Categorical variables were expressed as frequencies and percentages [n (%)] and compared between groups using the χ^2^ test. Univariate and multivariate logistic regression analyses were conducted with the occurrence of lower limb DVT within 21 days post-surgery (occurrence = 1, non-occurrence = 0) as the dependent variable to identify independent risk factors for lower limb DVT after PFNA surgery in elderly patients with IFF. To avoid overfitting given the limited number of DVT events (n = 43), we followed a conservative variable selection strategy. Variables with P < 0.10 in univariate analysis were considered for the multivariate model. The final model included four predictors: GLED use, age, BMI, and Evans classification (IV-V vs. III). The events-per-variable (EPV) ratio was 8.0, which is within acceptable limits for logistic regression. Finally, we stratified patients according to (1) Age: <75 years (n = 126) vs. ≥75 years (n = 101), (2) BMI: <24 kg/m^2^ (normal/underweight, n = 119) vs. ≥24 kg/m^2^ (overweight/obese, n = 108), and (3) Evans classification: Type III (less severe, n = 112) vs. Types IV-V (more severe/unstable, n = 115). Within each subgroup, we compared DVT incidence (days 8–14 and 15–21 combined, as the protective effect was most pronounced in these periods) between the conventional and GLED groups using Fisher’s exact test. Interaction P-values were calculated to assess whether treatment effect differed across subgroups.

## 3 Results

### 3.1 Baseline information

The baseline information between the conventional group and the GLED group showed no significant differences in age, gender ratio, BMI, smoking history, drinking history, etc. (P > 0.05; Table 1). Regarding comorbidities such as diabetes and hypertension, and fracture-related factors including Evans classification and injury cause, there were also no statistically significant differences observed between the two groups (P > 0.05). For surgical-related indicators, including operation time and intraoperative blood loss, no significant differences were observed between the two groups either (P > 0.05). These results indicate that the two groups are comparable in terms of baseline characteristics.

**TABLE 1:** Comparison of baseline information between two groups.

Parameter	Conventional group (n = 126)	GLED group (n = 101)	t/χ^2^	P
Demographics				
Age (years)	74.28 ± 5.65	74.13 ± 5.46	0.201	0.841
Gender [n (%)]	​	​	0.100	0.752
Male	50 (39.68%)	38 (37.62%)	​	​
Female	76 (60.32%)	63 (62.38%)	​	​
BMI (kg/m^2^)	24.51 ± 3.19	24.63 ± 3.04	0.287	0.774
Smoking [n (%)]	32 (25.40%)	24 (23.76%)	0.081	0.777
Drinking [n (%)]	25 (19.84%)	19 (18.81%)	0.038	0.845
Comorbidity				
Diabetes mellitus [n (%)]	32 (25.40%)	26 (25.74%)	0.004	0.953
Hypertension [n (%)]	57 (45.24%)	44 (43.56%)	0.064	0.801
Fracture-related				
Evans classification [n (%)]	​	​	0.051	0.975
III	63 (50.00%)	49 (48.51%)	​	​
IV	41 (32.54%)	34 (33.66%)	​	​
V	22 (17.46%)	18 (17.82%)	​	​
Cause of injury [n (%)]	​	​	0.967	0.326
Low-energy fall	120 (95.24%)	93 (92.08%)	​	​
High-energy trauma	6 (4.76%)	8 (7.92%)	​	​
Surgery-related				
Surgery time (min)	74.85 ± 10.62	73.29 ± 10.44	1.106	0.270
Intraoperative blood loss (mL)	161.43 ± 30.48	158.67 ± 29.25	0.690	0.491

GLED, ginkgo leaf extract and dipyridamole; BMI, body mass index.

### 3.2 Lower limb DVT

When comparing the incidence of postoperative lower extremity DVT between the two groups, it was found that there was no significant difference in the DVT incidence rate from immediate postoperative to 7 days (P = 0.386; Table 2). From day 8 to day 14 after surgery, the DVT incidence rate in the GLED group was significantly lower than that in the conventional group (P = 0.047). During the period from day 15 to day 21 after surgery, the DVT incidence rate in the GLED group was also significantly lower than that in the conventional group (P = 0.007). These results indicate that during the middle and late stages after surgery, the use of GLED can significantly reduce the risk of lower extremity DVT in patients.

**TABLE 2:** Comparison of the incidence of lower limb DVT between two groups [n (%)].

Parameter	Conventional group (n = 126)	GLED group (n = 101)	χ^2^	P
Postoperative immediate to 7 days	8/126 (6.35%)	3/101 (2.97%)	0.752	0.386
Postoperative 8∼14 d	15/118 (12.71%)	4/98 (4.08%)	3.952	0.047
Postoperative 15∼21 d	12/103 (11.65%)	1/94 (1.06%)	7.302	0.007

GLED, Ginkgo Leaf Extract and Dipyridamole; DVT, Deep Vein Thrombosis.

### 3.3 Coagulation parameters

The coagulation parameters between the two groups showed no significant differences in APTT, PT, FIB, and D-D levels at 8 h postoperatively (P > 0.05; Table 3). At 7 days postoperatively, the APTT in the GLED group was significantly higher than that in the conventional group (P = 0.020), and PT showed a similar trend, with the GLED group being significantly higher than the conventional group (P = 0.012). For FIB, the level in the GLED group at 7 days postoperatively was significantly lower than that in the conventional group (P < 0.001). The D-D levels in the GLED group were also significantly lower than those in the conventional group (P < 0.001). Two-way repeated-measures ANOVA revealed significant Group × Time interactions for all coagulation parameters (APTT: F = 5.82, P = 0.017; PT: F = 6.31, P = 0.013; FIB: F = 28.45, P < 0.001; D-D: F = 15.70, P < 0.001). These results indicate that within 1 week after surgery, the use of GLED leads to an extension of APTT and PT, while reducing FIB and D-D levels, suggesting that GLED may have an anticoagulant effect, which could help reduce the risk of thrombosis.

**TABLE 3:** Comparison of coagulation parameters between two groups.

Parameter	Conventional group (n = 126)	GLED group (n = 101)	t	P
APTT (s)				
Postoperative 8 h	30.55 ± 1.52	30.48 ± 1.49	0.355	0.723
Postoperative 7 d	31.24 ± 1.61	31.75 ± 1.67	2.343	0.020
PT (s)				
Postoperative 8 h	12.35 ± 0.62	12.33 ± 0.65	0.164	0.870
Postoperative 7 d	13.52 ± 0.68	13.75 ± 0.72	2.535	0.012
FIB (g/L)				
Postoperative 8 h	4.89 ± 0.75	4.86 ± 0.73	0.306	0.76
Postoperative 7 d	3.85 ± 0.66	3.18 ± 0.54	8.419	<0.001
D-D (μg/L)				
Postoperative 8 h	485.36 ± 83.15	478.92 ± 80.47	0.588	0.557
Postoperative 7 d	338.74 ± 76.24	225.41 ± 65.38	11.848	<0.001

GLED, ginkgo leaf extract and dipyridamole; APTT, activated partial thromboplastin time; PT, prothrombin time; FIB, fibrinogen; D-D, D-Dimer.

### 3.4 Hemorheological parameters

The hemorheological parameters of the two groups showed no significant differences in LS, MS, HS, and PV at 8 h postoperatively (P > 0.05; Table 4). At 7 days postoperatively, the levels of LS (P < 0.001), MS (P < 0.001), HS (P = 0.004), and PV (P < 0.001) in the GLED group were all significantly lower than those in the conventional group. Two-way repeated-measures ANOVA revealed significant Group × Time interactions for all hemorheological parameters (LS: F = 32.15, P < 0.001; MS: F = 45.82, P < 0.001; HS: F = 8.93, P = 0.003; PV: F = 38.62, P < 0.001). These results indicate that the use of GLED can significantly reduce blood viscosity within 1 week after surgery, suggesting that GLED may help improve blood circulation and reduce the risk of complications associated with blood viscosity.

**TABLE 4:** Comparison of hemorheological parameters between two groups (mPas).

Parameter	Conventional group (n = 126)	GLED group (n = 101)	t	P
LS				
Postoperative 8 h	18.53 ± 1.84	18.44 ± 1.81	0.369	0.713
Postoperative 7 d	15.26 ± 1.62	13.14 ± 1.47	10.241	<0.001
MS				
Postoperative 8 h	8.65 ± 0.78	8.61 ± 0.76	0.391	0.696
Postoperative 7 d	6.72 ± 0.53	5.66 ± 0.48	15.630	<0.001
HS				
Postoperative 8 h	5.24 ± 0.45	5.21 ± 0.43	0.426	0.670
Postoperative 7 d	4.87 ± 0.41	4.73 ± 0.32	2.944	0.004
PV				
Postoperative 8 h	1.86 ± 0.16	1.83 ± 0.15	1.619	0.107
Postoperative 7 d	1.63 ± 0.13	1.45 ± 0.11	11.234	<0.001

GLED, ginkgo leaf extract and dipyridamole; LS, low shear viscosity; MS, medium shear viscosity; HS, high shear viscosity; PV, plasma viscosity.

### 3.5 Inflammatory markers

**FIGURE 2:**
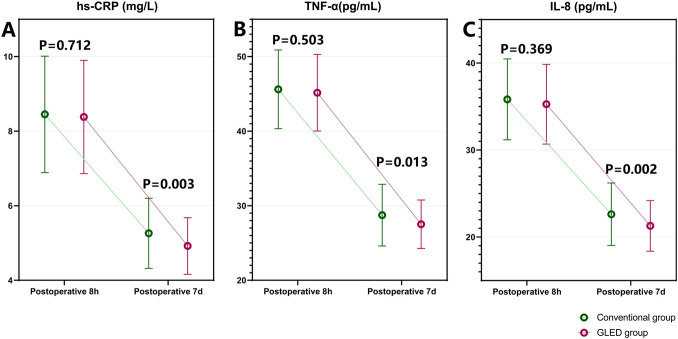
Comparison of inflammatory markers between two groups. **(A)** hs-CRP (mg/L); **(B)** TNF-α (pg/mL); **(C)** IL-8 (pg/mL). GLED, Ginkgo Leaf Extract and Dipyridamole; hs-CRP, high-sensitivity C-Reactive Protein; TNF-α, Tumor Necrosis Factor-alpha; IL-8, Interleukin-8.

When comparing the inflammation markers between the two groups, there were no significant differences in the levels of hs-CRP, TNF-α, and IL-8 at 8 h postoperatively (P > 0.05; Figure 2). At 7 days postoperatively, the levels of hs-CRP (P = 0.003), TNF-α (P = 0.013), and IL-8 (P = 0.002) in the GLED group were significantly lower than those in the conventional group. Two-way repeated-measures ANOVA revealed significant Group × Time interactions for all inflammatory markers (hs-CRP: F = 9.12, P = 0.003; TNF-α: F = 6.58, P = 0.011; IL-8: F = 10.24, P = 0.002). These results suggest that GLED may have anti-inflammatory effects, which could help reduce postoperative inflammatory responses, thereby promoting recovery and reducing the risk of complications.

### 3.6 Adverse reactions

The incidence of anticoagulation therapy-related adverse reactions between the two groups showed no significant difference in the overall incidence of adverse reactions between the conventional group and the GLED group (P = 0.209; Table 5). Although the incidence of gum bleeding, skin ecchymosis, and nausea and vomiting in the GLED group was slightly higher than that in the conventional group, none of these differences reached statistical significance. This indicates that GLED has acceptable safety during its application.

**TABLE 5:** Comparison of the occurrence of anticoagulation-related adverse reactions between two groups [n (%)].

Parameter	Conventional group (n = 126)	GLED group (n = 101)	χ^2^	P
The incidence of any adverse reaction	15 (11.90%)	18 (17.82%)	1.580	0.209
Gingival bleeding	8 (6.35%)	12 (11.88%)	​	​
Skin ecchymosis	10 (7.94%)	14 (13.86%)	​	​
Nausea and vomiting	5 (3.97%)	9 (8.91%)	​	​

GLED, ginkgo leaf extract and dipyridamole.

### 3.7 Logistic regression analysis

In the univariate regression analysis of risk factors for lower extremity DVT in elderly patients with IFF after PFNA surgery, the use of GLED was found to be a significant protective factor (P < 0.001, OR = 0.301), indicating its ability to reduce the risk of DVT (Table 6) significantly. Age (P = 0.012, OR = 1.051) and BMI (P = 0.008, OR = 1.083) were all identified as risk factors, meaning that for each unit increase in these indicators, the risk of DVT also increases. Additionally, Evans classification IV-V compared to III (P = 0.016, OR = 1.822) was also significant risk factors, suggesting that the higher the severity of the fracture, the greater the risk of DVT.

**TABLE 6:** Univariate regression analysis of risk factors for lower limb DVT after PFNA surgery in elderly patients with IFF.

Parameters	Coefficient	Std error	Wald	P	OR	95% CI
GLED	−1.200	0.350	11.755	<0.001	0.301	0.152–0.598
Age	0.055	0.022	6.250	0.012	1.051	1.011–1.093
BMI	0.085	0.031	7.519	0.008	1.083	1.021–1.149
Evans classification (IV-V vs. III)	0.650	0.253	6.498	0.016	1.822	1.116–2.974

PFNA, proximal femoral nail antirotation; IFF, intertrochanteric femoral fracture; GLED, ginkgo leaf extract and dipyridamole; BMI, body mass index; OR, odds ratio; CI, confidence interval.

In the multivariate logistic regression analysis of risk factors for lower extremity DVT in elderly patients with IFF after PFNA surgery, the use of GLED was identified as a significant protective factor (P = 0.001, OR = 0.307), indicating its capability to reduce the risk of DVT (Table 7) significantly. Age (P = 0.024, OR = 1.049) and BMI (P = 0.019, OR = 1.078) were all risk factors, meaning that for each unit increase in these indicators, the risk of DVT also increases. Evans classification IV-V compared to III (P = 0.026, OR = 1.786) was also a significant risk factor, suggesting that more severe fracture types increase the risk of DVT.

**TABLE 7:** Multivariate logistic regression analysis of risk factors for lower limb DVT after PFNA surgery in elderly patients with IFF.

Parameters	Coefficient	Std error	Wald stat	P	OR	OR CI lower	OR CI upper
GLED	−1.180	0.360	10.745	0.001	0.307	0.152	0.623
Age	0.048	0.021	5.224	0.024	1.049	1.007	1.093
BMI	0.075	0.032	5.493	0.019	1.078	1.012	1.148
Evans classification (IV-V vs. III)	0.628	0.260	4.976	0.026	1.786	1.073	2.973

DVT, deep vein thrombosis; PFNA, proximal femoral nail antirotation; IFF, intertrochanteric femoral fracture; GLED, ginkgo leaf extract and dipyridamole; BMI, body mass index; OR, odds ratio; CI, confidence interval.

### 3.8 Subgroup analysis

To assess whether the protective effect of GLED varies across different risk strata, we performed subgroup analyses stratified by age (<75 vs. ≥75 years), BMI (<24 vs. ≥24 kg/m^2^), and Evans classification (Type III vs. Types IV-V). As shown in Table 8, GLED reduced DVT incidence across all subgroups. The absolute risk reduction was larger in patients aged ≥75 years (20.60% vs. 12.73%), those with BMI ≥24 kg/m^2^ (19.30% vs. 14.05%), and those with Evans Types IV-V fractures (20.90% vs. 10.22%). The protective effect reached statistical significance only in the Evans Types IV-V subgroup (OR = 0.306, 95% CI 0.098-0.976, P = 0.041). Interaction P-values were non-significant for all three stratifications (P > 0.05), indicating no definitive evidence of effect modification. Nevertheless, the consistent trend toward larger absolute risk reductions in higher-risk subgroups suggests that patients with advanced age, higher BMI, or unstable fractures may derive particular benefit from GLED adjuvant therapy.

**TABLE 8:** Subgroup analysis of GLED efficacy on DVT incidence (postoperative days 8–21) by age, BMI, and Evans classification.

Subgroup	Conventional group (DVT/total)	GLED group (DVT/total)	DVT incidence reduction (%)	OR (95% CI)	P	Interaction P
Age					​	0.482
<75 years	11/68 (16.18%)	2/58 (3.45%)	12.73%	0.277 (0.053, 1.336)	0.108	​
≥75 years	16/58 (27.59%)	3/43 (6.98%)	20.61%	0.297 (0.088, 1.095)	0.058	​
BMI					​	0.873
<24 kg/m2	12/67 (17.91%)	2/52 (3.85%)	14.05%	0.267 (0.051, 1.284)	0.117	​
≥24 kg/m2	15/59 (25.42%)	3/49 (6.12%)	19.30%	0.283 (0.076, 1.072)	0.052	​
Evans classification					​	0.215
Type III	7/58 (12.07%)	1/54 (1.85%)	10.22%	0.251 (0.034, 2.332)	0.365	​
Types IV-V	20/68 (29.41%)	4/47 (8.51%)	20.90%	0.306 (0.098, 0.976)	0.041	​

GLED, ginkgo leaf extract and dipyridamole; DVT, deep vein thrombosis; BMI, body mass index; OR, odds ratio; CI, confidence interval.

## 4 Discussion

This study provides clinical evidence of an association between the combined use of GLED with standard rivaroxaban prophylaxis and a reduced incidence of lower extremity DVT. The findings indicate that this combined approach not only reduces the incidence of DVT, particularly during the subacute postoperative phase, but also modulates key pathological pathways associated with thrombosis, including the coagulation cascade, hemorheology, and systemic inflammation, without increasing minor bleeding complications.

One of the most striking findings of this study is the observed reduction in lower extremity DVT incidence in the GLED group during the intermediate (8–14 days postoperatively) and late (15–21 days postoperatively) phases. This temporal pattern suggests that while standard rivaroxaban treatment may effectively alleviate early postoperative hypercoagulability, the addition of GLED provides sustained protection during the later recovery stages when patients may have increased mobility but remain at risk due to ongoing endothelial dysfunction and hemorheological abnormalities (Li et al., 2025). The protective effects of GLED are consistent with previous findings showing synergistic reductions in thrombotic events when antiplatelet agents are combined with anticoagulants in orthopedic and cardiovascular surgeries (Sibbing et al., 2024). Particularly, the observed reduction in DVT incidence in this high-risk elderly population underscores the potential of GLED to fill gaps left by monotherapy and provide a more comprehensive preventive strategy.

The observed coagulation data suggest potential mechanistic pathways that may explain the antithrombotic effects of GLED. The observed prolongation of APTT and PT in the GLED group at 7 days postoperatively indicates its anticoagulant effect. While rivaroxaban directly inhibits factor Xa, it has been reported that components of GLED, particularly dipyridamole, can enhance the antiplatelet effects of endogenous prostacyclin and inhibit phosphodiesterase, leading to elevated cyclic adenosine monophosphate (cAMP) levels, which may indirectly regulate coagulation factor activity and platelet-endothelium interactions (Hou et al., 2023). The reduction in FIB and D-D levels in the GLED group suggests a potent inhibition of fibrin formation and turnover (Prasertcharoensuk et al., 2024). Elevated FIB is a recognized independent risk factor for DVT, contributing to hypercoagulability and increased plasma viscosity (Fang et al., 2024). These results are consistent with known pharmacological actions of ginkgo biloba flavonoids, which have been reported to inhibit thrombin activity and reduce platelet adhesion (Boateng, 2023). Our findings extend these experimental observations to a clinical setting, indicating that GLED may be more effective than rivaroxaban alone in inhibiting overall thrombin burst and subsequent fibrin deposition.

The improvement in hemorheological parameters further supports the hemodynamic principles underlying the prevention of DVT. The reduction in whole blood viscosity and plasma viscosity at all shear rates implies an improvement in blood flow (Riccio et al., 2024). Elevated blood viscosity is a significant contributor to venous stasis, a key component of Virchow’s triad, which is particularly prominent in elderly patients with reduced mobility (Zhang et al., 2025). Ginkgo leaf extract is known for its rheological properties, primarily attributed to its flavonoid content, which can reduce red blood cell aggregation and enhance deformability (Zhu and Liu, 2024). Dipyridamole also contributes to vasodilation and may improve the flexibility of red blood cells (Marco-Bonilla et al., 2023). Our findings are consistent with studies of GLED in cerebrovascular diseases, where its primary indication is to improve microcirculation (Wu et al., 2023). By effectively reducing blood viscosity, GLED may improve slow flow in the deep venous system of immobilized limbs, thereby reducing the stasis component of thrombosis. This action complements the anticoagulant effects of rivaroxaban, addressing different aspects of thrombotic risk.

The anti-inflammatory effects of GLED provide another potential mechanism for its beneficial actions. Post-traumatic and postoperative inflammation are powerful drivers of coagulation. Inflammatory cytokines such as TNF-α and IL-8 can activate endothelial cells, inducing a procoagulant phenotype through tissue factor expression, downregulation of thrombomodulin, and inhibition of fibrinolysis (Krajcsir et al., 2024). The observed reduction in levels of these markers suggests that GLED may mitigate the postoperative inflammatory cascade, thereby indirectly reducing the procoagulant environment (Asiwe et al., 2024). Ginkgo leaf extract possesses potent antioxidant and anti-inflammatory properties, inhibiting NF-κB signaling and the release of various inflammatory mediators (Salam et al., 2025). Dipyridamole has also shown anti-inflammatory effects in vascular models. By mitigating systemic inflammation surges, GLED may help protect endothelial integrity, inhibit inflammation-coagulation crosstalk, and thus create an environment less prone to thrombosis (Chen et al., 2024). This aligns with emerging literature positioning inflammation resolution as a therapeutic target for thromboprophylaxis (Li et al., 2023). Our findings add clinical weight to this concept, indicating that drugs with known anti-inflammatory properties can be associated with reduced inflammatory markers and lower DVT rates.

The three mechanistic axes described above—anticoagulation, hemorheological improvement, and anti-inflammation—are not independent but rather function synergistically to counteract the multifactorial pathophysiology of DVT (Virchow’s triad: hypercoagulability, stasis, and endothelial injury). First, the anti-inflammatory effect of GLED (reduction of hs-CRP, TNF-α, and IL-8) directly suppresses the inflammation-induced upregulation of tissue factor on endothelial cells and monocytes, thereby attenuating the extrinsic coagulation cascade. This creates a less prothrombotic milieu, which complements the direct anticoagulant actions of GLED (prolongation of APTT and PT, reduction of FIB and D-D). Second, the reduction in blood viscosity and improvement in red blood cell deformability (hemorheological effects) enhance venous return, particularly in the deep veins of the lower extremities. Improved flow not only reduces stasis (a primary risk factor for DVT) but also limits the local accumulation of activated coagulation factors and inflammatory cytokines, preventing the positive feedback loop between stasis, inflammation, and hypercoagulability. Third, by lowering systemic inflammatory markers, GLED may protect endothelial glycocalyx integrity and reduce leukocyte-endothelial adhesion, which further decreases microvascular resistance and improves rheology. In summary, GLED breaks the vicious cycle of ‘inflammation → hypercoagulability → microvascular dysfunction → stasis → thrombosis’ through simultaneous, mutually reinforcing actions on all three components of Virchow’s triad. This multimodal synergy explains why the addition of GLED to rivaroxaban provided superior DVT protection compared to rivaroxaban alone, especially in the subacute phase when inflammatory and rheological abnormalities are most prominent.

Safety analysis showed that the addition of GLED did not lead to an increase in anticoagulation-related adverse reactions. Although the GLED group exhibited numerically higher incidences of minor bleeding tendencies such as gum bleeding and bruising; considering its antiplatelet and vasodilatory effects, this is a predictable outcome. For the elderly population, balancing the benefits of DVT prevention through anticoagulation against the risk of bleeding is crucial (Lyu and Xu, 2024). This indicates that adjunctive use of GLED under monitored conditions does not pose unacceptable bleeding risks compared to standard rivaroxaban treatment.

Logistic regression analysis strongly positions GLED as an independent protective factor for DVT, even after adjusting for other significant risk factors such as age, BMI, fracture severity, and longer operation time. The robust protective odds ratio of GLED highlights its potential clinical utility in a multifactorial risk model. These results are consistent with existing literature, where studies have confirmed that advanced age, higher BMI, and more severe fracture types increase thrombotic risk (Zeng et al., 2025). Identifying these risk factors underscores the importance of individualized prevention strategies, suggesting that high-risk patients may particularly benefit from the addition of GLED to standard anticoagulation therapy.

Subgroup analyses revealed that the absolute risk reduction of DVT with GLED was numerically greater in patients aged ≥75 years, those with BMI ≥24 kg/m^2^, and those with Evans Type IV-V fractures, although interaction tests were not significant. Notably, the benefit was statistically significant only in the unstable fracture subgroup (Types IV-V). This finding aligns with the pathophysiological understanding that more severe fractures induce greater inflammatory and hypercoagulable responses, potentially creating more room for multimodal interventions like GLED. These results support the individualized use of GLED in high-risk elderly patients with unstable intertrochanteric fractures. However, given the limited sample size within subgroups and the absence of significant interactions, these findings should be considered hypothesis-generating and require confirmation in larger prospective trials.

Compared with its individual components, the GLED regimen demonstrates clear synergistic advantages. Dipyridamole monotherapy has historically shown limited efficacy as a DVT prophylactic agent. In a controlled trial by Dechavanne et al., the combination of dipyridamole and acetylsalicylic acid failed to reduce DVT incidence (50% vs. 40% in controls), while a subsequent double-blind study by Silvergleid et al. found that dipyridamole plus ASA was ineffective in elective total hip replacement (thrombosis rate 36% in the treated group vs. 25% in controls) (Dechavanne et al., 1975; Silvergleid et al., 1978). Ginkgo leaf extract monotherapy possesses antiplatelet and rheological properties but, as a single agent, its antithrombotic efficacy in orthopedic DVT prevention remains insufficiently established. In contrast, the GLED combined regimen addresses all three components of Virchow’s triad through multi-target mechanisms: Ginkgo leaf extract provides anti-inflammatory and hemorheological improvement, while dipyridamole offers antiplatelet aggregation and vasodilation. A network meta-analysis of 13 TCM injections combined with LMWH for orthopedic DVT prevention found that GLED ranked third (87.0%) in lowering FIB levels, a key hypercoagulability marker, while demonstrating an acceptable safety profile (Zeng et al., 2021). Another meta-analysis confirmed that integrated traditional Chinese and western medicine therapy significantly reduced DVT incidence after lower extremity orthopedic surgery (RR = 0.40, 95% CI 0.30–0.54, P < 0.00001) (Zhu et al., 2018). Collectively, GLED provides balanced, multimodal protection against DVT, making it a valuable adjunctive option for elderly patients with elevated fibrinogen levels and significant inflammatory responses.

Despite these promising findings, our study has several limitations worth considering. First, this study has limitations in the collection of baseline variables. Although Table 1 shows that the two groups were balanced in common variables such as age, gender, BMI, major comorbidities, and fracture types, we did not systematically collect and report other recognized important confounding factors that could influence DVT risk. These factors include: the interval from injury to surgery, preoperative bed rest duration, time to first postoperative ambulation, American Society of Anesthesiologists classification, Charlson Comorbidity Index, preoperative mobility, type of anesthesia, and nutritional indicators such as hemoglobin and albumin levels, particularly a history of prior venous thromboembolism and malignancy. The absence of these variables is a common limitation in single-center retrospective studies. If these unmeasured factors are unevenly distributed between the two groups, they may constitute residual confounding, affecting the accuracy of our estimation of GLED efficacy. However, it is worth considering that in clinical practice, physicians are more likely to prescribe GLED for patients perceived to have higher thrombotic risk (e.g., poorer preoperative mobility, more comorbidities, longer surgical delays). If such “confounding by indication” exists, it suggests that the baseline thrombotic risk in the GLED group may be higher than in the conventional group. In this case, the significantly lower DVT incidence observed in the GLED group might actually underestimate the true protective effect of GLED. Nonetheless, we cannot quantify the magnitude of this potential bias. Future prospective studies should predefine and systematically collect all these important covariates at the design stage to allow for more thorough adjustment in analyses, thereby leading to more robust conclusions. Second, no *a priori* sample size estimation was performed, which is inherent to the retrospective design. Although *post hoc* power analysis for the primary DVT outcome indicated adequate power (0.82), the lack of pre-specified sample size remains a limitation. Future prospective studies should include formal sample size calculations. Third, the DVT follow-up assessment in this study was only conducted up to postoperative day 21, failing to cover the complete 30-day medication treatment period. Although this captured the highest-risk period, asymptomatic or late-onset DVT occurring between days 22 and 30 may have been missed, potentially underestimating the true incidence of DVT. Future studies should extend ultrasound monitoring to at least 30 days or longer. Fourth, at the level of mechanism exploration, this study only measured conventional inflammatory markers such as hs-CRP, TNF-α, and IL-8. Although the results showed that GLED could reduce the levels of these markers, to more thoroughly elucidate the specific molecular mechanisms by which it regulates the “cross-talk between inflammation and coagulation,” future studies should measure additional key molecules directly involved in this process. These include pro-inflammatory core cytokine interleukin-6, the primary initiator of the coagulation process tissue factor, and thrombomodulin, a key regulatory protein for endothelial anticoagulant function. Analysis of these markers would help more precisely reveal how GLED affects endothelial function, platelet activation, and the coagulation cascade, thereby completing the puzzle of its multi-target mechanism at the molecular level. Fifth, DVT was assessed at fixed time points rather than continuously, resulting in interval-censored data. While we used period-specific incident risks as the primary analysis, time-to-event methods such as Cox regression would be more appropriate in prospective studies with exact event dates. Future studies should record the exact date of DVT diagnosis to enable survival analysis. Sixth, while we performed repeated-measures ANOVA to assess group-by-time interactions, the primary analysis relied on separate t-tests at each time point. This approach does not fully account for within-subject correlations. However, the additional repeated-measures analyses confirmed the robustness of our findings. Future studies with more than two time points should employ linear mixed models to better characterize temporal trajectories.

## 5 Conclusion

This study demonstrates that adjunctive use of GLED on the basis of standard rivaroxaban anticoagulation can reduce the incidence of lower extremity DVT in elderly patients undergoing PFNA surgery for intertrochanteric fractures during the intermediate and long-term postoperative periods. Its protective effect may be related to a multi-target mechanism involving synergistic enhancement of anticoagulation, reduction of blood viscosity, and inhibition of systemic inflammatory response.

This study suggests that adjunctive use of GLED Injection on the basis of standard rivaroxaban anticoagulation is associated with a lower incidence of lower extremity DVT in elderly patients undergoing PFNA surgery for intertrochanteric fractures. However, these findings are derived from a single-center retrospective observational study and should be considered hypothesis-generating. Prospective randomized controlled trials are needed to establish causality.

## Data Availability

The original contributions presented in the study are included in the article/supplementary material, further inquiries can be directed to the corresponding author.
